# Advances in the Molecular Biology of Chondrosarcoma for Drug Discovery and Precision Medicine

**DOI:** 10.3390/cancers17162689

**Published:** 2025-08-19

**Authors:** Robert Lee Walker, Francis J. Hornicek, Zhenfeng Duan

**Affiliations:** Department of Orthopedic Surgery, Sarcoma Biology Laboratory, Sylvester Comprehensive Cancer Center, University of Miami Miller School of Medicine, Papanicolaou Cancer Research Building, 1550 NW, 10th Avenue, Miami, FL 33136, USA; rlw153@miami.edu (R.L.W.); fjh21@med.miami.edu (F.J.H.)

**Keywords:** chondrosarcoma, molecular pathogenesis, precision medicine, tumor microenvironment, targeted therapy

## Abstract

Chondrosarcomas (CSs) are generally slow-growing tumors of cartilage-producing cells, and the second most common primary bone malignancy following osteosarcoma. CSs are typically resistant to conventional chemo- and radiotherapy, and aggressive surgical resection with wide margins remains the only effective treatment option. Immunotherapies and targeted therapies in CSs have failed in clinical trials, and no prognostic biomarkers exist within the clinic. Although CSs have undergone histologic and genetic analysis, the molecular mechanisms that drive their pathogenesis and resistance are still largely unknown. A deeper understanding of the molecular biology and dysregulated pathways in CSs is essential for more efficient precision therapies. Several integrated large-scale genetic and epigenetic studies have recently been reported in CS, with several distinguished pathways holding therapeutic promise. In this review, we summarize the advances in the molecular biology of CSs, focusing on both genomic and epigenomic mechanisms as well as potential biomarkers and targets. These studies highlight several valuable clinical opportunities for earlier detection, prognostic accuracy, and therapeutic targeting that may improve patient outcomes in CSs.

## Simple Summary

Chondrosarcoma is a rare bone cancer that often resists chemotherapy and radiation, making it difficult to treat. In this study, we explore the genetic, epigenetic, and immune-related changes that occur in different subtypes of chondrosarcoma. By analyzing how specific gene mutations and signaling pathways contribute to tumor development and progression, we aim to identify new molecular targets that could be used for more effective treatments. We also highlight how the tumor’s cellular environment may play a role in patient outcomes and response to therapy. Our goal is to provide a comprehensive overview that could help researchers and clinicians design personalized treatment strategies and improve future drug development for this challenging disease.

## 1. Introduction

Chondrosarcomas (CSs) represent a heterogeneous group of malignant bone tumors that likely originate from mesenchymal stem cells committed to the chondrocytic lineage. These tumors exhibit slow growth and are embedded within a distinct tumor microenvironment (TME) rich in hyaline cartilaginous neoplastic matrix [1,2]. CSs are the second most frequently diagnosed primary bone malignancy after osteosarcoma (OS), comprising approximately 20–30% of all malignant bone tumors [3,4]. In contrast to OS, which typically affects children and adolescents, CSs predominantly arise in adults, particularly in the hip and femur [5].

Chondrosarcomas (CSs) are categorized as either primary (conventional) or secondary tumors. Primary CSs develop de novo within normal bone, whereas secondary CSs evolve from preexisting benign cartilage lesions such as enchondromas or osteochondromas [6]. The most common form of primary CSs is conventional chondrosarcomas (CHSs), which make up most cases (85–90%). CHSs are divided into 3 subtypes—central, periosteal, and peripheral. The remaining cases are made up of nonconventional CSs, which include clear cell, mesenchymal, and dedifferentiated subtypes [7]. CSs are also graded on a histological basis, which accounts for nuclear size, hyperchromasia, cellularity, pleomorphism, and mitoses [8,9,10]. Grade 1 (G1, low-grade) or atypical cartilaginous tumor (ACT) is defined by poor cellularity, small hyperchromatic nuclei, and a lack of miotic figures [8]. These CSs rarely metastasize and are considered aggressive neoplasms rather than malignant sarcomas. The 5-year survival rate for grade 1 CSs is 85–95% [9,10]. Grade 2 (G2, intermediate grade) is comparatively more cellular to grade 1, while also showing nuclear enlargement, and rare miotic activity [8]. These CSs have intermediate metastatic potential when compared to grade 1 and grade 3 tumors. The 5-year survival rate for grade 2 CSs is 70–85% [9,10]. Grade 3 (G3, high-grade) tumors are hypercellular with nuclear pleomorphism and show increased miotic activity and necrosis. These CSs have high metastatic potential, which has resulted in a 5-year survival rate of less than 20% [8,9,10]. With the advancement of diagnostic tools and sequencing techniques, there is a growing interest in understanding the molecular drivers and therapeutic vulnerabilities of chondrosarcoma subtypes to inform treatment decisions.

For most cases of low-grade CS, wide surgical resection usually results in an adequate outcome due to its slow growth and rare metastasis [11]. Thus, surgical resection has remained the primary treatment modality for these cases. However, patients with high-grade (advanced, metastatic, or unresectable) CS often show a dismal prognosis and a short median survival time of less than 12 months. High-grade CS is often lethal, recurrent, and metastatic to the lung, liver, kidney, and brain [2,12]. This poor outcome is primarily due to the intrinsic resistance of high-grade CS to conventional chemotherapy and radiotherapy. In addition, substantial resistance to multiple investigated targeted and immunotherapies has been reported [12,13].

Differentiating between low- and high-grade CS is complicated by the absence of both predictive and prognostic biomarkers [14]. Limited treatment strategies within the clinic have resulted in a plateau of outcomes over several decades. There is an urgent need for novel CS treatment options. A deeper understanding of the molecular mechanisms and pathway dysregulation in CS is essential for identifying actionable therapeutic targets. This review summarizes recent advances in the molecular biology of CS, with a focus on genomic and epigenomic alterations, potential biomarkers, and emerging therapeutic targets, while highlighting how these findings may inform the development of precision treatment strategies for this aggressive malignancy.

## 2. Amplified or Deleted Genes in Chondrosarcoma

Copy number variations (CNVs) are structural alterations in the genome characterized by the duplication or deletion of DNA segments relative to the normal genome. In cancer, CNVs usually lead to an increased expression of oncogenes or the loss of tumor suppressor genes. Pinpointing gene amplifications or deletions in cancer genomes is essential for understanding the drivers of cancer initiation and progression, revealing biomarkers that may aid in diagnosis and prognosis, and identifying potential therapeutic targets (Table 1).

CNVs can be detected using various techniques, including comparative genomic hybridization (CGH) microarrays, sequencing-based approaches such as next-generation sequencing (NGS), and PCR-based methods like digital PCR. Several CGH studies have been pivotal in revealing CNVs that frequently arise in CS. In one CGH study utilizing 15 samples of high-grade CS, amplification at 8q24.12-q24.13 was identified as the most frequent genetic alteration [15]. Notably, this region is associated with the MYC oncogene. Utilizing a validation arm of 116 cartilaginous tumors, MYC amplification was observed in G2 (15%), G3 (20%), and dedifferentiated (21%) CSs. However, no amplification was observed in enchondromas nor in G1 CSs. c-Myc expression was observed in all samples where MYC was amplified and in samples without detectable MYC DNA amplification. In these cases, polysomy 8 was frequently observed, occurring in 18% of G1, 31% of G2, and 80% of G3, and 29% of dedifferentiated CSs, but not in enchondromas. Statistical analysis revealed a correlation between shorter overall survival time with CS grade, polysomy 8, and MYC amplification [15]. These CGH findings highlight the potential of MYC amplification and polysomy 8 as prognostic biomarkers in CS. Further studies are needed to validate the role of MYC in CS development and its potential as a therapeutic target.

In another CGH study, analysis of 21 CS tumor samples revealed CNVs. Genomic alterations were primarily observed in G3 samples. Analysis of all samples revealed 22 imbalanced chromosome regions in ≥25% of samples, with amplification occurring on 3 regions on chromosome 12 (12p13, 12p11.21-p11.23, and 12q13) [16]. The gain of 12q13 has been reported in CS and other sarcomas [17,18]. Importantly, genes found in this region have been associated with tumorigenesis and include sarcoma amplified sequence (SAS), cyclin-dependent kinase 4 (CDK4), and glioma-associated oncogene homolog (GLI) (Table 1). RNA expression analysis revealed higher levels of CDK4 expression in CS tumors where 12q13 was amplified [16]. Notably, CDK4 has been associated with malignant metastasis and poor prognosis in CS patients and has been identified as a potential therapeutic target in other sarcomas [19,20]. Amplification of 12p11.21-p11.23 was also observed. Genes in this region include parathyroid hormone-like hormone (PTHLH) and PTPRF-interacting protein-binding protein 1 (PPFIBP1) (Table 1) [16]. PTHLH has been reported to be expressed in all enchondromas and CSs, likely due to its role in chondrocyte growth and differentiation [21,22]. PPFIBP1 has been shown to interact with the calcium-binding protein S100A4, which is associated with tumor invasiveness and metastasis [23]. This study also reported that amplification of chromosome 12 alongside loss of chromosome 6 was associated with high-grade CS. This unique pair of genetic alterations could be a prognostic biomarker in high-grade CS and possibly identify specific genetic changes not found in lower grades. Deletion of 9p21.3-p24.1 was also observed and previously reported in another study of CS [16,17]. This region contains the INK4A/INK4A-ARF locus, which encodes two tumor suppressor genes via alternative reading frames: p16INK4A (CDKN2A, encoding the p16 protein) and p14ARF (also from CDKN2A, encoding the p14 protein) (Table 1). Loss of this locus results in loss of heterozygosity (LOH), a consistent genetic alteration in conventional CS that is also associated with high-grade CS [24,25]. In another investigation using CS tumor samples, a correlation between INK4A/p16 expression and tumor grade was observed. Together with the retention of these proteins in enchondromas, this suggests that loss of INK4A/p16 expression is likely an important event in the progression from enchondroma to conventional CS, as well as in histological grade progression after recurrence [24]. Revisiting the previous study, deletion of 9p was associated with the ribosomal protein S6 (RPS6) gene (Table 1) [16]. Low expression of RPS6 correlated with 9p deletion and was found in high-grade CS tumors. RPS6 contributes to the control of cell growth and proliferation through selective translation of certain classes of mRNA and is hypothesized to be a candidate tumor suppressor gene in CS [16,26]. Deletions in chromosome 1 were also detected, specifically, 1p13.2-p22.1 and 1p36.22-p36.31 [16]. These deletions have also been reported in a previous CS study [17]. The 1p13.2-p22.1 region contains exostosin-like glycosyltransferase 2 (EXTL2). This gene is homologous to exostosin glycosyltransferase 1/2 (EXT1 and EXT2), and initiates heparan sulfate synthesis (Table 1). EXT1/2 encode for glycosyltransferases involved in heparan sulfate sidechain elongation. Mutations in EXT1/2 have been implicated in osteochondroma formation; however, secondary peripheral chondrosarcomas often retain functional EXT alleles, suggesting that malignant progression may involve EXT-independent mechanisms [27]. Histologically, conventional central CS resembles secondary peripheral CS; thus, EXTL2 may be a target for deletion whose absence may distinguish similarities and differences between these CS subtypes [16].

More recently, investigations have employed NGS to uncover genetic alterations characteristic of CS. In an NGS study of dedifferentiated CS, a patient with a recurrent tumor initially exhibited a TP53 frameshift mutation. Recurrence occurred after radiation therapy, and the tumor displayed an increased proliferation index. Subsequent NGS analysis revealed the persistence of the TP53 mutation along with PTEN deletion, present in both the cartilaginous and noncartilaginous components of the tumor. PTEN deletion was associated with reduced PTEN DNA copy number and lower protein expression. A second patient with dedifferentiated CS, who displayed neither the TP53 mutation or the PTEN deletion, nor recurrence, was used as a control. The authors concluded that PTEN loss in the background of TP53 mutation could underlie the increased proliferative capacity in the recurrent tumor [28]. These findings provide further insight into the deletions observed in CS. Interestingly, the combination of TP53 mutation and PTEN deletion may be characteristic of recurrent CS and could serve as prognostic biomarkers pending further investigation (Table 1).

HIF-2α is a transcription factor encoded by the gene endothelial PAS domain-containing protein 1 (EPAS1) and is responsible for modulating the hypoxic response like HIF-1α (Table 1). Both HIF-1α and HIF-2α play distinct roles in regulating hypoxic genes and tumor progression. Amplification of HIF1A does not significantly impact overall or disease-free survival outcomes in patients with CS. However, amplification of EPAS1 has been reported to be associated with decreased overall survival and increased dedifferentiation in CS patients [29]. EPAS1 amplification induces high expression of HIF-2α, which is commonly observed in high-grade CS. Notably, HIF-2α is associated with drug resistance in CS and is a downstream effector of Sirtuin 1 (SIRT1) (Table 1) [29]. SIRT1 is a nuclear-localized deacetylase that requires nicotinamide adenine dinucleotide (NAD^+^) as a cofactor for its enzymatic activity. It can deacetylate histone and non-histone proteins, allowing it to influence various regulatory pathways such as the nuclear factor-κB family, TP53 family members, and forkhead box (FOXO) transcription factors [30]. Through its regulation of key signaling pathways and transcription factors, SIRT1 influences cellular energy metabolism, promotes epithelial-to-mesenchymal transition (EMT), and contributes to metastatic progression. Compared to low-grade CSs, SIRT1 is highly expressed in high-grade and dedifferentiated CSs and is correlated with poor prognosis in CS patients. SIRT1 poses a promising therapeutic target, as silencing it has led to the in vivo inhibition of tumor growth in CS cell lines [31].

## 3. Mutated Genes and Aberrant Signaling Pathways in Chondrosarcoma

**Figure 1:**
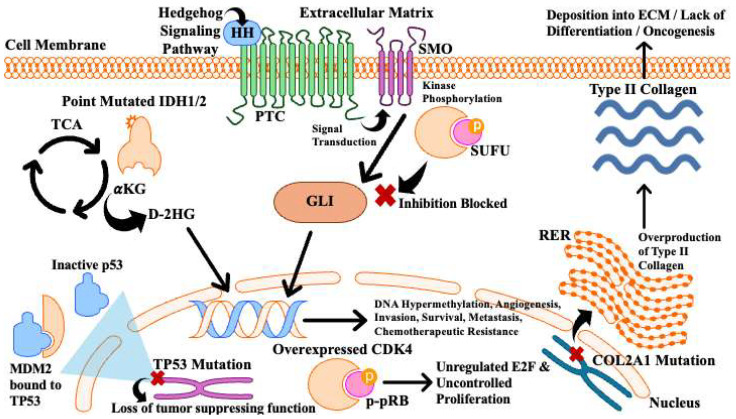
Key Mutated Genes and Aberrant Signaling Pathways Driving Chondrosarcoma Progression. This schematic illustrates the major genetic mutations and dysregulated signaling pathways implicated in CS development and progression. Mutations in TP53 lead to inactivation of p53, impairing tumor suppressor functions, which may be further inhibited by aberrantly expressed MDM2. Overexpression of CDK4 results in pRB phosphorylation (p-pRB), releasing E2F to drive uncontrolled cell cycle progression. Mutations in COL2A1 cause excessive type II collagen production, contributing to extracellular matrix (ECM) accumulation and impaired chondrocyte differentiation—hallmarks of CS oncogenesis. Point mutations in IDH1/2 alter tricarboxylic acid (TCA) cycle metabolism, producing the oncometabolite D-2-hydroxyglutarate (D-2HG) from α-ketoglutarate (αKG). D-2HG accumulation leads to HIF-1α overexpression, promoting glycolytic gene expression, angiogenesis, and invasion. Additionally, aberrant Hedgehog (HH) signaling via dysregulated PTCH1, SMO, and GLI activation enhances transcription of genes that support tumor survival, metastasis, and therapeutic resistance. These interconnected genetic and pathway alterations collectively contribute to the aggressive biology of CS.

Genetic mutations are a common hallmark of cancer and often serve as the initial trigger for tumor initiation and progression. Several somatic and germline mutations have been identified in CS [2,32]. Many mutated genes can contribute to the development and growth of CS (Figure 1 and Table 1).

### 3.1. IDH1/2

IDH1/2 mutations are the most frequently occurring mutations in CS and are key contributors to malignant transformation among other genetic abnormalities (Table 1). Among the various chondrosarcoma subtypes, IDH mutations have been reported in approximately 87% of enchondromas, 50% of central conventional chondrosarcomas, and more than 80% of dedifferentiated chondrosarcomas [33]. A mutation analysis of 25 high-grade CSs revealed that 61% of cases harbored a somatic IDH1/2 mutation, with most cases (86%) containing an IDH1 mutation [34]. In another study utilizing primary and recurrent CS tissue samples, IDH mutations were detected in primary tumors, as well as in locally recurrent and metastatic lesions [35]. IDH1/2 mutation analysis may serve as a distinguishing biomarker between CS and chondroblastic osteosarcoma [36]. Notably, the point mutations in IDH1/2 found in CS often differ from those observed in other tumors, including acute myeloid leukemia (AML) and glioma. CS predominantly harbors R132C mutations in IDH1, whereas gliomas typically exhibit R132H mutations in IDH1, and AML commonly presents R140Q mutations in IDH2. However, since these mutations are not entirely exclusive to specific cancers, they may reflect shared tumorigenic pathways across distinct tumor types [36].

IDH1/2 catalyzes the oxidative decarboxylation of isocitrate into α-ketoglutarate (aKG). However, IDH1/2 point mutations induce substitution at R132 in IDH1, and R140 or R172 in IDH2, which disrupts the natural function of IDH in the cell. Mutated IDH1/2 will convert aKG into an oncometabolite known as D-2-hydroxyglutarate (D-2HG), a competitive inhibitor of aKG-dependent enzymes (Figure 1) [37,38,39]. D-2HG inhibits histone demethylases, tet methylcytosine dioxygenase 2 (TET2), and hypoxia-inducible factor (HIF) prolyl hydroxylases. The inhibition of these enzymes promotes histone and DNA hypermethylation, thereby repressing the expression of genes essential for chondrocyte differentiation. Consequently, D-2HG drives chromatin dysregulation and epigenetic changes that contribute to the initiation and early progression of CS [11]. D-2HG also induces overexpression of HIF-1α, thereby enhancing the transcriptional activity of its target genes. Many of these genes are involved in angiogenesis, invasion, and glucose metabolism–processes essential for tumor growth and development. Overexpression of HIF-1α and glycolysis-associated genes has been reported in high-grade CSs [11]. Given the frequency of IDH1/2 mutation in CS and its role in early tumor development, IDH1/2 represents both a distinguishing biomarker and a potential therapeutic target.

### 3.2. TP53

In cancer, TP53 is one of the most frequently mutated genes, and its pathway is pivotal for the control of cell cycle progression and apoptosis. In CS, TP53 is the second most frequently altered gene, occurring in approximately 22% of cases, and is predominantly found in high-grade and dedifferentiated subtypes (Table 1) [8,40,41,42]. Correlations between TP53 alterations or overexpression and tumor histological grade and metastasis in CS have been reported [43,44]. In a cohort of chondrosarcoma patients, TP53 mutations were linked to significantly poorer overall survival, shorter metastasis-free intervals, and a heightened risk of both local recurrence and metastatic relapse [40]. Notably, TP53 genetic alterations are typically absent in well-differentiated tumors, supporting their association with more aggressive forms of CS [41].

Mutation of TP53 results in the loss of crucial tumor suppressor function, which significantly contributes to the malignant progression of CS (Figure 1). LOH in the 17p13 region, leading to TP53 loss, occurs in approximately 25–30% of high-grade CSs [45,46]. Additionally, mutations in both intronic and exonic regions of TP53 have been reported [47]. Amplification of the 12q13 region, which contains the mouse double minute 2 homolog (MDM2), is also observed in high-grade CSs. Binding between TP53 and MDM2 induces TP53 inactivation (Figure 1) [32]. In one study, MDM2 overexpression was detected in 33% of high-grade chondrosarcomas and was positively associated with higher histological grades, as demonstrated by immunohistochemistry (IHC) [41]. Targeting the TP53-MDM2 interaction may offer a strategy to restore normal TP53 functionality. Moreover, studies suggest that TP53 status could help stratify CS patients who are at higher risk of relapse or disease progression [11].

### 3.3. RB1

The retinoblastoma susceptibility gene (RB1) is a tumor suppressor gene that encodes the pRB protein, which prevents cell cycle progression from the G1 to S phase by binding and inhibiting the transcription factor E2F. Regulation of pRB is mediated by CDK4/6, which phosphorylate pRB, suppressing its activity and thereby promoting E2F activation and cell cycle progression [48]. Overexpression and aberrant activity of CDK4 have been reported in CS, where they disrupt cell cycle regulation and lead to uncontrolled proliferation (Table 1 and Figure 1) [19].

Complete deletion or low expression of RB1 has frequently been observed in high-grade CS (Table 1). One study reported aberrant RB pathway activity in 96% of high-grade CS cases, driven by decreased levels of p16 (48%), increased levels of CKD4 (55%), or elevated cyclin D1 levels (62%) [49]. The complete deletion or reduced expression of RB1 may serve as a biomarker for CS progression and prognosis. Moreover, CDK4 represents a potential therapeutic target that could mitigate the effects of RB1 loss or restore pRB function. CDK4 inhibitors have demonstrated efficacy in other cancers, including sarcomas such as osteosarcoma [48].

### 3.4. COL2A1

COL2A1 encodes the α-chain of type II collagen fibers, which is synthesized by chondrocytes and is a key component of articular cartilage [50]. Type II collagen constitutes 90–95% of the collagen in the extracellular matrix (ECM) [51]. In a comparative whole-exome sequencing (WES) study conducted by the Cancer Genome Project on 49 chondrosarcoma cases, researchers identified 1428 somatic mutations, with individual tumors exhibiting mutation burdens ranging from 1 to 115. These mutations included 944 missense, 61 nonsense, 37 essential splice-site, 80 indel, and 301 synonymous changes. Notably, COL2A1 mutations, in the form of insertions, deletions, and rearrangements, were identified in 37% of cases. These findings suggest that COL2A1 mutations disrupt normal collagen biosynthesis in CS (Table 1) [42].

CS is an ECM-rich sarcoma, in which collagen is a major component; it is likely that COL2A1 mutations drive aberrant collagen production (Figure 1) [42,52,53]. Alterations in ECM composition and signaling may promote tumor progression by disrupting collagen differentiation, contributing to oncogenesis [54]. Consequently, COL2A1 mutation in CS may represent a hallmark alteration of matrix deposition and signaling, making it a potential therapeutic target.

### 3.5. Hedgehog

The Hedgehog (HH) signaling pathway is essential for chondrocyte proliferation and bone development (Table 1) [55]. Three Hedgehog-related genes have been identified: sonic hedgehog (SHH), desert hedgehog (DHH), and Indian hedgehog (IHH). Following production, HH proteins undergo post-translational modification, during which they are cleaved into active signaling molecules. These proteins are then secreted and bind to a membrane receptor, patched (PTC). HH ligand binding to PTC relieves its inhibition of the membrane protein smoothened (SMO), thereby activating the signaling cascade. The signal is transduced to the nucleus via the GLI family of transcription factors, which are normally inhibited by the scaffolding protein suppressor of fused (SUFU). However, aberrant HH signaling leads to the phosphorylation of SUFU, allowing GLI proteins to enter the nucleus and regulate transcription (Figure 1) [55]. In CS, dysregulation of this pathway promotes various cellular responses, including enhanced survival and altered differentiation [56].

WES analysis of CS revealed that 18% of tumors harbor mutations in HH signaling genes [42]. Notably, high-grade CSs exhibit elevated expression levels of HH pathway components. Mutations in SHH-associated genes, including HH receptor PTCH1 and transcription factors GLI2/3, can constitutively activate HH signaling. This activation contributes to the formation of benign cartilage-based tumors, including enchondroma, osteochondroma, chondroblastoma, periosteal chondroma, and chondromyxoid fibroma [42,57,58]. Although these neoplasms are benign, they can serve as precursor lesions to malignant CS.

Chondrogenesis is regulated by the IHH/parathyroid hormone-related protein (PTHrP) pathway, in which IHH stimulates chondrocyte proliferation, while PTHrP delays terminal differentiation through a regulatory feedback mechanism. Given the critical role of HH signaling in chondrogenesis, therapeutic targeting of this pathway in CS is being explored. SMO inhibitors, such as HPI-4 and IPI-929, have demonstrated preclinical efficacy in disrupting aberrant HH pathway activity in CS models [59,60,61]. Additional HH pathway-related mutations reported in CS include PTCH1 mutations, inactivating mutations in SUFU, and GLI1 amplifications [42].

### 3.6. TGF-β1 Signaling

The transforming growth factor beta (TGF-β) superfamily consists of a range of conserved genes that encode ligands and receptors interacting with SMAD transcription factors to regulate gene transcription [62]. In mesenchymal CS (MSC), high levels of phosphorylated SMAD2 (p-SMAD2) have been observed in the tumor’s small cell components. Additionally, p-SMAD1 and plasminogen activator inhibitor 1 (PAI-1) are highly expressed in the cartilaginous components of MSC tumors. TGF-β signaling is also involved in the regulation and maintenance of Sox9, a master regulator of chondrogenic differentiation [63]. Notably, p-SMAD2 directly interacts with TGF-β, underscoring the pathway’s relevance in MSC biology. These findings suggest that TGF-β signaling may play a critical role in MSC pathogenesis and highlight this pathway as a potential source of therapeutic targets [63,64].

## 4. Fusion Genes in Chondrosarcoma

Although rare compared to DNA amplifications, deletions, or mutations, certain CS subtypes harbor and express fusion genes. In MCS, two recurrent fusion genes have been identified: HEY1-NCOA2 and IRF2BP2-CDX1 (Table 1) [65,66].

### 4.1. HEY1-NCOA2

Most cases of MSC contain the HEY1-NCOA2 fusion, resulting from an intrachromosomal deletion of 8q [64,67]. Recent studies using an MSC mouse model have provided insights into the biological function of this fusion gene [68]. In these experiments, HEY1-NCOA2 was introduced into mouse embryonic superficial zone (eSZ) cells, which were then subcutaneously transplanted into nude mice. The expression of HEY1-NCOA2 induced tumor formation in approximately 69% of mice, with tumors displaying the characteristic biphasic morphology of human MSC. This suggests HEY1-NCOA2 is a potent oncogenic driver in the development of MSC. Chromatin immunoprecipitation sequencing (ChIP-seq) demonstrated that HEY1-NCOA2 binding overlapped with H3K27ac peaks at active enhancers of key genes including Sox9, Runx2, Runx3, and Hes1, indicating HEY1-NCOA2 drives a cartilage-specific transcriptional program through enhancer activation [68].

HEY1, a component of the Notch signaling pathway, typically regulates targets like Hes1. However, when fused with NCOA2, HEY1-NCOA2 no longer operates canonically within Notch signaling, though it still modulates Notch/HEY1 targets such as Hes1 [68,69,70,71]. HEY1 has also been shown to reduce COL2A1 expression levels, affecting its interaction with Sox9 [68,72]. Through NCOA2, HEY1-NCOA2 recruits transcriptional machinery such as histone acetyltransferases, promoting H3K27 acetylation. Importantly, a regulatory axis between HEY1-NCOA2 and Runx2 was identified: CRISPR-mediated knockout of Runx2 delayed tumor onset in vivo but resulted in poorly differentiated tumors dominated by small round cells. Notably, Runx2-deficient tumors exhibited accelerated growth, suggesting that Runx2 balances proliferation and differentiation in MCS [68]. The functional role of HEY1-NCOA2 was further explored using the histone deacetylase (HDAC) inhibitor panobinostat, which inhibited HEY1-NCOA2-associated gene expression (Sox9, Runx1, Hes1) and tumor growth in xenograft models. These findings highlight the role of epigenetic regulation in MSC pathogenesis and suggest that HDAC inhibitors may offer a promising therapeutic strategy [68]. Altogether, HEY1-NCOA2 plays a central role in MSC development, differentiation, and proliferation, and understanding its regulatory pathways could inform future therapeutic approaches.

### 4.2. IRF2BP2-CDX1

IRF2BP2-CDX1 is another fusion gene found in MSC (Table 1). Although less common than HEY1-NCOA2, this fusion gene likely results from an intrachromosomal rearrangement involving the long arm of chromosome 8. Specifically, the translocation associated with this fusion is t(1;5) (q42;q32), which fuses exon 1 of the IRF2BP2 gene on chromosome 1 with intron 1 of the CDX1 gene on chromosome 5 [66]. IRF2BP2 encodes a protein containing a zinc finger motif, enabling DNA binding. Notably, IRF2BP2 interacts with TP53 and the oncogene IRF2, implicating it in tumorigenesis [73,74]. CDX1 is a transcription factor with irregular expression observed in intestinal cancer [75,76,77]. While IRF2BP2-CDX1 is associated with MSC, its functional role remains poorly understood.

## 5. Epigenetic Alterations in Chondrosarcoma

Epigenetics refers to the modification of DNA and chromatin that regulates gene expression without altering the DNA sequence, while maintaining miotic stability. Advances in molecular biology have established epigenetic dysregulation as a hallmark of various cancers [78]. Accordingly, recent studies have identified epigenetic alterations—particularly DNA methylation and ncRNAs—as key contributors to CS development [79].

### 5.1. DNA Methylation

DNA methylation, catalyzed by DNA methyltransferases (DNMTs), involves the addition of methyl groups to DNA and is essential for regulating gene expression [79,80]. Aberrant DNA methylation, particularly hypermethylation of promoter CpG islands, can silence tumor suppressor genes and is a common feature in cancer [81]. In CS, hypermethylation of genes such as p16 and Runx3 has been reported, suggesting their potential as prognostic indicators [79]. Additionally, hypermethylation of the Wnt inhibitory factor 1 (WIF1) gene has been observed in CS cell lines (CS-1 and SW1353) and tumor tissues [82].

WIF1 inhibits Wnt proteins, a family of cysteine-rich glycoproteins implicated in cancer pathogenesis [83]. By binding Wnt ligands, WIF1 prevents activation of the canonical Wnt/β-catenin signaling pathway, which involves the frizzled receptor and low-density lipoprotein receptor-related proteins 5/6 (LRP5/6). Activation of this pathway normally triggers the intracellular protein disheveled (Dvl), leading to β-catenin stabilization and the transcription of oncogenes via the TCF/LEF complex [84,85]. WIF1 suppresses this cascade by blocking Wnt ligand binding, promoting β-catenin degradation, and inhibiting oncogenic transcription.

In CS, loss of WIF1 expression due to hypermethylation results in dysregulated Wnt signaling. This has been documented across various sarcoma subtypes, including CS [82,86]. One study demonstrated that loss of WIF1 correlates with increased expression of Wnt pathway proteins, including Wnt5a/b, LRP6, and Dvl2, as shown by Western blot analysis in CS samples. Notably, elevated methylation levels of WIF1 were significantly correlated with reduced overall and progression-free survival in patients with chondrosarcoma [82].

As previously discussed, IDH1/2 mutations play a key role in CS development. When mutated, IDH enzymes produce the oncometabolite D-2HG, which inhibits aKG-dependent enzymes. This inhibition leads to DNA hypermethylation and histone modifications, both of which have been implicated in CS pathogenesis [87]. Hypermethylation driven by IDH mutations has been linked to genes involved in stem cell maintenance and lineage differentiation. Among these, several have emerged as potential therapeutic targets, including TET enzymes, Aurora kinases, and HDACs [88].

Beyond IDH-driven effects, several other genes have been reported to undergo hypermethylation in CS, including p16INK4a, E-cadherin, and nicotinamide phosphoribosyltransferase (NAMPT). Inhibitors of NAMPT and nicotinate phosphoribosyltransferase (NAPRT) have shown potential efficacy in high-grade CS [89]. Runx3 is also subject to hypermethylation in CS, and it has been shown to inhibit proliferation and induce apoptosis in CS cells [90]. Similarly, hypermethylation of p73, a member of the p53 family, has been correlated with CS histological grade and progression. One study demonstrated that loss of p73 expression correlates with increased methylation of its promoter, and that treatment of CS cell lines with a DNA demethylating agent can restore p73 expression [91]. These findings suggest that p73 may serve as both a prognostic marker and a therapeutic target in CS.

### 5.2. Non-Coding RNAs

Non-coding RNAs (ncRNAs) are functional RNA molecules that do not encode proteins but instead regulate gene expression, often through epigenetic mechanisms. Key classes of ncRNAs include microRNAs (miRNAs), long non-coding RNAs (lncRNAs), and circular RNAs (circRNAs)

The role of miRNAs in cancer has been extensively studied, revealing their critical involvement in tumor progression. miRNAs regulate gene expression by binding to the 3′ untranslated region (3′ UTR) of target mRNAs. They also contribute to normal chondrogenesis; for instance, miR-140 regulates HDAC4 to promote chondrocyte hypertrophy via Runx2 [41]. In CS, several miRNAs are dysregulated, including miR-30a, miR-100, miR-145, miR-181a, and miR-221 [57,58,92,93]. Dysregulation of miRNAs in CS influences oncogenesis. For example, miR-100 is typically downregulated in CS and normally functions as a tumor suppressor by targeting overexpressed mTOR. Similarly, miR-30 inhibits tumor growth via SOX4 suppression but is reduced in CS, while miR-181a is upregulated and promotes CS progression by enhancing VEGF expression [41].

lncRNAs also play significant roles in CS. The lncRNA HOTAIR is upregulated in CS tissues and cell lines, with elevated levels correlating with advanced tumor stage and poor prognosis. Knockdown of HOTAIR has been shown to inhibit CS growth both in vitro and in vivo [94]. Another lncRNA, BCAR4, is also upregulated in CS tissues and cell lines [95]. BCAR4 epigenetically activates the mTOR signaling pathway by inducing hyperacetylation of histone H3, promoting CS cell proliferation and migration. In vivo studies have demonstrated that BCAR4 overexpression accelerates CS tumor growth, while knockdown suppresses it [94].

Together, these findings suggest that lncRNAs such as BCAR4, along with other ncRNA subtypes, contribute to CS pathogenesis. Given their regulatory influence on key oncogenic pathways, ncRNAs present promising targets for therapeutic intervention, warranting further investigation to fully elucidate their roles in CS.

## 6. Approaches for In-Depth Analysis and Comprehensive Understanding of Chondrosarcoma

### 6.1. Integrated Multi-Omics and Multi-Layered Profiling in Chondrosarcoma

Recent studies have highlighted the genomic complexity and heterogeneity of CS, encompassing variations in DNA mutations, structural alterations, copy number changes, epigenetic modifications such as DNA methylation, and gene expression profiles. Despite these advances, the precise molecular mechanisms and regulatory networks underlying CS initiation and progression remain incompletely understood. Integrated multi-omics approaches, which combine diverse omics technologies—including genomics, epigenomics, transcriptomics, proteomics, and metabolomics—generate multilayered data that provide a more holistic understanding of disease biology, particularly in cancer [96]. As biotechnology advances, the elusive behaviors of tumors like CS are becoming increasingly decipherable.

Due to the rarity of CS, integrated multi-omics analyses that simultaneously examine whole-genome sequencing, epigenetics, and gene expression data (including DNA, RNA, and methylation alterations) from the same tumor tissue remain uncommon. One study, however, utilized mRNA expression, miRNA profiles, and DNA methylation data to propose a multi-omic signature capable of stratifying high-risk CS patients [54]. This study, leveraging genetically characterized CS cohorts, identified a spectrum of genetic abnormalities associated with an aggressive CS phenotype and poor prognosis. Key findings included silencing of the 14q32 imprinted locus—leading to downregulation of several miRNAs—and genome-wide DNA hypermethylation induced by IDH mutations [54].

The proposed multi-omic signature delineated prognostic categories: patients with a favorable prognosis exhibited either IDH wild-type with high 14q32 expression (IDHwt/14q32high), IDH-mutant with high 14q32 expression (IDHmut/14q32high), or IDH wild-type with low 14q32 expression (IDHwt/14q32low). Intermediate prognosis was associated with IDH-mutant/14q32low expression and/or high proliferative activity. The poorest prognosis, characterized by dedifferentiated CS, was defined by low 14q32 expression coupled with high proliferation. Analysis of relapsed samples further suggested that CS tumors may acquire increasingly aggressive features over time, although the precise timing of such transformations remains unclear [54]. Importantly, this study demonstrated that an integrated molecular evaluation using multi-omics provides a more comprehensive and reliable prognostic assessment of CS than IDH status alone [11].

### 6.2. RNA Sequencing Approaches for Transcriptomic Profiling of Chondrosarcoma

The transcriptome comprises coding messenger RNAs (mRNAs) as well as non-coding RNAs (ncRNAs), including microRNAs (miRNAs), long non-coding RNAs (lncRNAs), ribosomal RNAs (rRNAs), and transfer RNAs (tRNAs). While various techniques have been developed to study these RNAs in human disease, next-generation sequencing (NGS) has emerged as the primary platform for the comprehensive and unbiased profiling of cancer genomes and transcriptomes. RNA sequencing (RNA-Seq) is a high-throughput technology that provides a detailed view of the entire transcriptome, enabling isoform detection, gene fusion identification, gene expression profiling, targeted sequencing, and single-cell analysis.

In another study on CS, single-cell RNA sequencing (scRNA-seq) was employed to distinguish transcriptional signatures based on proliferation, stromal, and leukocyte-related genes [97]. Through the application of a proliferation index and an immunosuppression index, researchers were able to differentiate high-grade and dedifferentiated tumors, while an active immune response index was typically associated with low-grade tumors. Additional markers served as prognostic indicators; for instance, endoplasmic reticulum (ER) stress regulators, such as DNA damage-inducible transcript 3 (DDIT3) and heat shock protein family A member 5 (HSPA5), correlated with overall survival in conventional CS patients. Elevated expression of DDIT3 or HSPA5 has been associated with poorer prognosis in chondrosarcoma. Moreover, in a patient-derived xenograft (PDX) model, endoplasmic reticulum (ER) stress induction promoted tumor growth, whereas pharmacological inhibition of ER stress suppressed tumor progression [97]. These findings highlight the potential of ER stress regulators as therapeutic targets and underscore the value of integrating transcriptomic analyses within multi-omics approaches to gain a comprehensive understanding of CS initiation, progression, and metastasis.

Transcriptomic data integration has also been used to explore mechanisms of doxorubicin resistance in CS [98]. This study found that CS cells significantly secreted hepatocyte growth factor (HGF), and knockdown of both HGF and its receptor MET enhanced doxorubicin sensitivity in CS cells. Analysis of the GEO database, RNA-Seq, and Gene Set Enrichment Analysis (GSEA) further confirmed that HGF and MET expression levels were significantly elevated in CS tissues compared to normal cartilage [98].

## 7. In Vitro Preclinical Modeling of Chondrosarcoma

### 7.1. Chondrosarcoma Cell Lines

Cancer cell lines are valuable tools for in vitro studies, offering versatile applications in preclinical research. However, establishing cancer cell lines can be challenging, particularly for malignancies such as chondrosarcoma (CS), which typically exhibits slow growth. Additionally, there is a scarcity of cartilage-derived cell lines that retain a stable cartilage phenotype [99,100,101]. Currently, 82 confirmed human CS cell lines have been reported [11]. Among these, the most widely cited include SW1353, JJ012, CH2879, OUMS-27, and HCS-2/8 [99,101,102,103]. These and other cell lines have been extensively employed across a range of CS studies. For example, SW1353 is frequently used for drug testing and signaling pathway studies, JJ012 is commonly utilized in assays related to invasion, metastasis, and radiosensitivity, and CH2879 is notable for its efficacy in establishing orthotopic models [19,103,104,105,106,107,108,109].

Novel cell lines continue to be developed, such as the recently established dedifferentiated CS cell line, DDCS2 [110]. Dedifferentiated CS is particularly aggressive, characterized by a higher risk of metastasis and poorer prognosis. While these cell lines provide useful experimental models, they do not fully recapitulate the clinical heterogeneity of chondrosarcoma, which encompasses diverse histological subtypes, genetic alterations, and resistance to conventional therapies [11].

### 7.2. Chondrosarcoma Organoid Models and Other 3D Modeling Techniques

The establishment of cancer cell lines is essential for studying various diseases, including chondrosarcoma (CS). However, many of these lines are cultured in vitro using two-dimensional (2D) systems, which lack the capacity to mimic the complex TME. This limitation is particularly problematic in CS, a malignancy with a notoriously hostile, ECM-rich TME that hinders drug penetration and efficacy. There remains a critical clinical need to develop new therapeutic strategies that are effective under these challenging conditions where conventional treatments fail.

Advancements in organoid and three-dimensional (3D) models have provided valuable tools for recapitulating the intricate and dynamic features of the TME. These models offer the advantage of more accurately predicting in vivo tumor behavior, generating robust data to enhance preclinical studies and potentially improve the design of clinical trials. Organoids are 3D in vitro systems derived from self-organizing cells capable of replicating the structural and functional characteristics of their tissue of origin. Establishing tumor organoids typically requires scaffold-based or scaffold-free methods to prevent direct contact with plastic surfaces; the most common scaffold is Matrigel, a gelatinous protein mixture derived from mouse sarcoma cells that mimics the ECM. Organoid models of CS have already been applied to study miRNA dynamics [111].

In a recent study, patient-derived tumor organoids (PDTOs) from sarcomas, including CS, were utilized to assess drug resistance and sensitivity. The findings demonstrated that PDTO drug sensitivity correlated with clinical features such as tumor subtype, treatment history, and disease trajectory. Notably, CS PDTOs exhibited a chemoresistant phenotype consistent with clinical observations, underscoring the importance of authentic models for advancing treatment strategies [112]. These insights highlight the value of PDTOs in supporting personalized medicine approaches and warrant further investigation into overcoming drug resistance in CS.

3D spheroid models of CS have also been developed, effectively replicating the chemoresistant and radioresistant properties of CS more authentically than 2D cultures [113,114,115]. For example, in one study, the poly (ADP-ribose) polymerase inhibitor talazoparib sensitized CS cells to chemotherapy and radiotherapy in 2D conditions. However, in 3D spheroids, a reduction in tumor growth was only observed after prolonged treatment, and radiotherapy was less effective overall, inhibiting growth in only one of three tested CS cell lines [115]. Another study combining CS 3D spheroids with monocytes revealed that the spheroids induced monocyte polarization into pro-tumoral M2 macrophages, which in turn enhanced spheroid growth, suggesting a pro-tumoral crosstalk within the TME [116].

Collectively, these studies illustrate the limitations of 2D cultures in replicating authentic tumor behavior. Three-dimensional modeling offers a more physiologically relevant environment for CS cells, providing a critical platform to test hypotheses and evaluate treatment strategies. The continued development of such models will be instrumental in generating translational data applicable to in vivo systems and clinical settings.

## 8. In Vivo Preclinical Modeling of Chondrosarcoma in Mice

### 8.1. Cell Line-Derived Xenograft Models of Chondrosarcoma

Cell line-derived xenograft (CDX) models involve the transplantation of pre-established cancer cell lines into mice, either subcutaneously or orthotopically. Several CS cell lines, including SW1353, CH2879, and JJ012, have been used to develop CDX models for chondrosarcoma.

In one study, SW1353 and CH2879 cells were transplanted both subcutaneously and orthotopically into mice to generate CDX models [109]. The CS cells were transduced with a lentiviral luciferase construct to enable longitudinal monitoring of tumor growth via bioluminescence imaging. Mice injected with either cell line were treated with doxorubicin over a six-week period. Tumor growth was observed in all mice, with CH2879 tumors growing larger than those formed by SW1353. Notably, there was no significant difference in tumor size between the subcutaneous and orthotopic groups. Importantly, doxorubicin treatment did not significantly inhibit in vivo tumor growth, underscoring the chemoresistant nature of CS [109]. Similarly to organoid and 3D culture models, these CDX models faithfully recapitulated the drug-resistant phenotype of CS.

In another study, the JJ012 cell line was used to establish an orthotopic CDX mouse model of CS [103]. Prior to in vivo experiments, JJ012 cells cultured in 3D conditions exhibited high proliferative capacity, invasion, and colony formation. Orthotopic transplantation of JJ012 into mice generated tumors localized to intratibial and periosteal sites. Periosteal tumors grew to three times the size of the contralateral, non-injected limb within seven weeks, while intratibial tumors reached a similar size by ten weeks. Histological analysis of tumor sections revealed features consistent with high-grade CS. Additionally, lung micrometastases were detected in all five periosteal tumor-bearing mice and in two of four intratibial tumor-bearing mice [103].

These findings demonstrate the utility of JJ012 in generating a reliable and clinically relevant CDX model of human CS. Of particular significance are the model’s ability to replicate local bone invasion and lung metastasis, key hallmarks of CS aggressiveness. Such models provide a valuable platform for evaluating novel therapeutic strategies and chemotherapeutic agents aimed at curbing CS progression and metastasis.

### 8.2. Genetically Engineered Xenograft Models of Chondrosarcoma

The HEY1-NCOA2 fusion gene is expressed in the majority of MSC cases. To elucidate its functional role in MSC development and progression, a previously discussed study transduced mouse embryonic superficial zone (eSZ) cells with HEY1-NCOA2 [68]. Following confirmation of fusion gene expression by RT-qPCR, the transduced cells were subcutaneously transplanted into BALB/c nude mice. Tumor formation occurred in approximately 69% of recipients, with tumors exhibiting biphasic morphology and Sox9 expression. ChIP sequencing revealed that HEY1-NCOA2 binds to several active enhancers and interacts with Runx2. Treatment with the histone deacetylase (HDAC) inhibitor panobinostat suppressed tumor growth both in vitro and in vivo, while also reducing expression of HEY1-NCOA2 and Runx2 [68].

This study demonstrates the utility of genetically engineered CS xenograft models for investigating the functional consequences of genetic alterations such as HEY1-NCOA2. The findings suggest that HEY1-NCOA2 hijacks and amplifies the chondrogenic differentiation transcriptional program, driving MSC development and progression [68].

### 8.3. Patient-Derived Xenograft Models of Chondrosarcoma

PDX models involve the direct transplantation of patient tumor tissue into immunocompromised or humanized mice. These models have been successful in recapitulating the characteristics of human cancers, including spatial architecture and intratumor heterogeneity. Importantly, PDX models can retain the genomic features of the original patient tumors across various disease stages, cancer subtypes, and treatment histories [117]. Although PDX models have proven highly valuable in cancer research, their application in CS remains limited, with relatively low transplantation success rates. Nevertheless, ongoing advancements in engraftment techniques may improve the feasibility of establishing CS PDX models [11].

A PDX model of MSC has been successfully developed using primary tumor and distant metastasis tissues from a patient. The transplanted tumors maintained expression of the HEY1-NCOA2 fusion gene. Treatment with imatinib, a tyrosine kinase inhibitor, significantly reduced tumor growth both in vitro and in the PDX model, demonstrating the potential of imatinib as an effective therapeutic agent for MSCs [118]. This study underscores the potential of CS PDX models to serve as reliable platforms for studying tumor biology and evaluating targeted therapies.

Other efforts to develop CS PDX models have yielded variable engraftment success rates. For instance, in one study, only 1 out of 10 subcutaneously implanted CS biopsies generated a viable tumor in nude mice that reflected the phenotype of the patient’s tumor [119]. In a separate study, 17 PDX models were generated from human CS tumor samples, though the study did not report specific engraftment rates or growth characteristics [120]. Several factors influence PDX engraftment success, including tumor type and characteristics, sample quality and handling, mouse strain, and implantation site. PDX models also offer advantages for studying CS tumor metabolism in vivo, avoiding the limitations of in vitro conditions.

In the collection of 17 CS PDX models, both IDH wild-type and IDH-mutant CS samples were represented. This included eight central CSs, seven dedifferentiated CSs, and two clear cell CSs. Metabolic profiling revealed distinct signatures between IDH wild-type and mutant tumors; notably, IDH-mutant tumors exhibited elevated levels of amino acids, lactate accumulation, and increased acylcarnitine concentrations. However, the study did not provide details regarding engraftment efficiency, growth kinetics, stabilization over passages, or model cryopreservation—information that is especially valuable given the rarity of CS PDX models. Incorporating molecularly targeted inhibitors into these models could further enhance their translational relevance for clinical applications [120].

Several CS PDX models are publicly or commercially available through repositories such as the National Cancer Institute (CF8X) and the PDXNet Consortium (WU-0064) [121]. Conventional CS models CTG-1255 and CTG-2383 can be obtained from Champions Oncology, while the Jackson Laboratory offers a panel of eight conventional CS models for drug screening studies [118,122]. Additionally, two conventional CS PDX models, CS-347 and CS-281, have been established; CS-347 represents an intermediate-grade tumor, while CS-281 is high-grade. Both models showed high expression of Ephrin type-A receptor 2 (EphA2). Treatment with the EphA2 inhibitor ALW II-41-27 resulted in dose-dependent growth inhibition in vitro, suggesting EphA2 as a promising therapeutic target in CS [123].

## 9. Potential Targeted Therapies and Treatment Strategies for Chondrosarcoma

To date, the Food and Drug Administration (FDA) has not approved any therapies for conventional CS or for advanced stages of the disease. This underscores the urgent need for effective treatment options, particularly for refractory, inoperable, and metastatic CS. Several targeted therapies have been proposed, including inhibitors of PI3K/Akt, mTOR, and PDGFR, but these have yielded disappointing results in clinical evaluations [2,41,124]. More recently, genomic studies have uncovered promising targetable genes and pathways that drive CS progression. Emerging targets such as IDH, Hedgehog signaling, Rb, and CDK4 have been shown to play pivotal roles in CS pathogenesis (Table 2). Further investigation into these and other molecular targets may provide the critical breakthroughs needed to advance CS therapy.

### 9.1. IDH

Under normal physiological conditions, IDH catalyzes the production of αKG. Mutant IDH, however, converts αKG to the oncometabolite D-2HG. Although the precise mechanisms by which mutant IDH drives CS remain partially understood, some studies have shed light on its functional impact and associated pathways. In one study, CRISPR/Cas9-mediated knockout of IDH1 in two CS cell lines led to significant reductions in D-2HG production, anchorage-independent growth, and cell migration. In vivo, IDH1 knockout in a xenograft model similarly resulted in decreased D-2HG levels. RNA sequencing of IDH1 knockout cells revealed downregulation of several integrin genes, suggesting that integrin-mediated processes are integral to the tumorigenicity driven by mutant IDH1 in CS cells [125]. This indicates that integrins could serve as promising co-targets alongside IDH1 in therapeutic strategies.

Notably, two IDH inhibitors—enasidenib (AG-221) and ivosidenib (AG-120)—have been approved by the FDA for treating IDH-mutant relapsed or refractory acute myeloid leukemia (AML) [38,126]. Ivosidenib is also approved for IDH1-mutated cholangiocarcinoma, while the dual IDH1/2 inhibitor vorasidenib has been approved for glioma [127,128]. Given this therapeutic potential, ivosidenib is currently undergoing clinical evaluation in CS. In a phase I clinical trial involving patients with IDH-mutated chondrosarcoma, treatment with ivosidenib resulted in a median progression-free survival (PFS) of 5 months. Stable disease was achieved in 52% of participants, although no objective responses—complete or partial—were reported during the initial two-year evaluation period (NCT04278781) (Table 2) [129].

A follow-up analysis revealed that patients maintaining disease stability for over two years exhibited an overall response rate of 23%. The data also indicated differential sensitivity to IDH inhibition between CS subtypes. Specifically, progression-free survival rates at 3 and 6 months were 30% and 0% for dedifferentiated CS, respectively, and 77% and 54% for conventional CS [130]. This suggests that, despite similar IDH mutation status, subtypes of CS may exhibit distinct responses to IDH-targeted therapies. A phase II trial evaluating ivosidenib in locally advanced, recurrent, or metastatic intermediate- to high-grade IDH1-mutant CS is currently ongoing (NCT04278781). Additionally, the CHONQUER study, a phase III clinical trial, is recruiting patients to assess oral ivosidenib in different IDH1-mutated CS, including locally advanced or metastatic conventional CS grades 1–3 that are ineligible for curative resection (NCT06127407) (Table 2).

Given the ongoing clinical evaluation of IDH inhibitors and the differential responses observed between CS subtypes, there is an urgent need to better stratify patients by IDH1/2 mutation status. Among the various candidate biomarkers identified in CS, IDH1/2 profiling represents the most immediately actionable and clinically needed. Accurate mutation detection may enhance therapeutic precision, identify patients most likely to benefit from IDH-targeted therapies, and inform treatment selection in both clinical trial settings and standard practice.

### 9.2. Hedgehog

The Hedgehog (HH) signaling pathway plays a critical role in the development and progression of CS. High levels of HH target genes, including PTCH1 and GLI1, are commonly expressed in CS tissues [60,131]. In preclinical models, dysregulated HH signaling has been shown to significantly impact CS cell proliferation. For example, elevated HH protein expression enhances CS proliferation, while pharmacological inhibition of HH signaling reduces proliferation rates in vitro [132].

In one study, CS xenograft models were established from 12 human tumor samples using NOD-SCID mice. Treatment with triparanol, an HH signaling inhibitor, resulted in a 60% reduction in tumor volume, a 30% decrease in cellularity, and a 20% reduction in proliferation rate within the xenografts [131]. Another HH pathway inhibitor, saridegib (IPI-926), is a potent, orally administered small molecule that targets SMO, a key transducer of HH signaling. In primary CS xenograft models, saridegib treatment led to downregulation of the HH pathway, evidenced by decreased expression of GLI1 and PTCH1, alongside reduced tumor growth (Table 2) [60].

Saridegib also disrupted both autocrine and paracrine HH signaling and induced histopathological changes in treated tumors, including increased calcification and tumor cell loss. Gene expression profiling following saridegib treatment revealed alterations in several genes, including ADAMTSL1, which is known to regulate CS proliferation (Table 2) [60].

These findings underscore the importance of the HH signaling pathway in CS pathogenesis and provide a strong rationale for its therapeutic targeting.

### 9.3. CDK4 and Rb

CDK4 and Rb are both upregulated in CS and are functionally linked via the CDK4/Rb signaling pathway, which plays a critical role in cell cycle regulation. Dysregulated CDK4 activity in CS correlates with genomic amplification at the 12q13 locus [16], and CDK4 overexpression is associated with metastasis and poor patient prognosis [16,19,49]. In CS cell lines, CDK4 knockdown via shRNA significantly reduced cell viability, proliferation, and clonogenic potential [19,49].

These findings have prompted investigations into CDK4-targeted therapies in CS. The CDK4 inhibitor palbociclib induces G1 phase cell cycle arrest and reduces cell migration and invasion by disrupting the CDK4/Rb pathway. In vivo, palbociclib treatment has been shown to decrease CS tumor burden [19]. Additionally, in a phase I dose-defining study, the heat shock protein 90 (HSP90) inhibitor alvespimycin achieved disease stabilization for more than six months in a CS patient, accompanied by a reduction in CDK4 expression (Table 2) [133].

Collectively, these results support CDK4 inhibition as a promising therapeutic strategy, particularly for patients with high-grade CS.

### 9.4. HIF-2α and SIRT1

In high-grade CS, hypoxia-inducible factor 2 alpha (HIF-2α) is overexpressed and associated with poor prognosis. In a study utilizing two CS xenograft models—generated by orthotopic or subcutaneous injection of SW1353 and JJ012 cells into mice—the HIF-2α small molecule inhibitor TC-S7009 significantly inhibited tumor growth. Moreover, combining TC-S7009 with either cisplatin or doxorubicin produced a synergistic antitumor effect, exceeding the efficacy of each monotherapy. These findings suggest that HIF-2α inhibition sensitizes CS cells to apoptosis, thereby enhancing the effectiveness of DNA-damaging agents such as doxorubicin. This combinatorial strategy highlights a promising therapeutic avenue, wherein HIF-2α inhibition may restore drug sensitivity in chemoresistant CS cells [29]. These data provide a rationale for incorporating HIF-2α inhibitors into combination treatment regimens for CS (Table 2).

Dysregulation of NAD^+^ metabolism is another hallmark of several cancers, including CS. While targeting NAD^+^ metabolism poses challenges due to its involvement in numerous cellular processes, many cancers—including CS—develop heightened NAD^+^ dependencies by upregulating associated metabolic pathways [11]. CS tumors with poor prognosis often display increased NAD^+^ biosynthesis, regulated in part by the SIRT1-HIF-2α axis. Consequently, inhibiting SIRT1 disrupts this axis, exposing vulnerabilities conferred by NAD^+^ dependence and impairing CS progression [31].

In one study, the SIRT1 inhibitor EX527 demonstrated significant antitumor synergy when combined with doxorubicin in CS xenograft models [31]. This combination effectively leveraged the disruption of NAD^+^ metabolism to enhance the cytotoxic effects of chemotherapy. These findings warrant further exploration of SIRT1 inhibition in CS, particularly in combination with standard chemotherapeutic agents, to overcome drug resistance and improve therapeutic outcomes (Table 2).

### 9.5. Death Receptor 5

Death receptor 5 (DR5) is a proapoptotic multimeric cell surface receptor that binds tumor necrosis factor-related apoptosis-inducing ligand (TRAIL). Upon binding TRAIL, DR5 acts as a potent inducer of apoptosis in tumor cells while sparing normal cells [134]. In one study, the recombinant TRAIL agonist Apo2L/TRAIL (dulanermin) was used to activate both DR4 and DR5, resulting in a strong and sustained antitumor effect in a patient with metastatic CS [135]. Currently, a phase I clinical trial is underway evaluating IGM-8444, a multimeric anti-DR5 IgM agonist antibody, in solid tumors, including CS (NCT04553692) (Table 2) [136].

Another DR5 agonistic antibody, INBRX-109, is a tetravalent single-domain antibody engineered to engage four DR5 receptors simultaneously, demonstrating superior antitumor activity. The development of INBRX-109 was prompted by its efficacy in preclinical studies targeting DR5 in two conventional CS PDX models, CTG-1255 and CTG-2383 [122]. INBRX-109 was evaluated in a phase I clinical trial that included solid tumors such as CS (NCT03715933). Notably, INBRX-109 showed antitumor activity irrespective of IDH1/2 mutation status, achieving a disease control rate of 87% (including stable disease and partial responses), with a median progression-free survival (PFS) of 7 months. This outcome was comparable to PFS with pazopanib and superior to that of regorafenib and ivosidenib in CS [122].

In a phase II clinical trial, INBRX-109 achieved a sustained clinical benefit in 40.7% of patients (11 of 27), including two partial responses (NCT03277924) [137]. A randomized, placebo-controlled phase II trial is currently planned to further evaluate INBRX-109 in patients with unresectable or metastatic conventional CS (NCT04950075) (Table 2).

### 9.6. Chondroitin Sulfate Proteoglycan 4

Chondroitin sulfate proteoglycan 4 (CSPG4) is highly expressed on the surface of CS tumor cells and exhibits minimal expression in normal tissues, making it an attractive therapeutic target [11]. In a study of 76 CS patients, 71% of conventional CS samples showed high CSPG4 expression, with the highest prevalence in grade 2 tumors. By contrast, only 15% of dedifferentiated CS samples expressed CSPG4. Elevated CSPG4 expression was associated with early metastasis in conventional CS and reduced overall survival in dedifferentiated CS [138].

In the same study, genetically engineered CSPG4-targeted chimeric antigen receptor T (CAR-T) cells effectively eliminated CS tumor cells in vitro [138]. Targeting CSPG4 holds considerable therapeutic promise, as CSPG4 inhibition has also demonstrated notable efficacy in osteosarcoma models [139]. Further investigation is warranted to explore the role of CSPG4 in CS progression and assess the potential of CSPG4-directed therapies in preclinical and clinical settings (Table 2).

### 9.7. Therapeutic Angiogenesis Inhibition

Vascular endothelial growth factor A (VEGF-A) drives angiogenesis in solid tumors. Tumor cells secrete VEGF-A, which binds to VEGFR-1 and VEGFR-2 and promotes tumor cell proliferation, migration, and survival [11]. VEGF-A overexpression has been observed in chondrosarcoma (CS) [140].

Pazopanib, a multi-targeted tyrosine kinase inhibitor, inhibits several angiogenesis-related receptors, including VEGFRs, platelet-derived growth factor receptors (PDGFRs), and KIT. In a phase II clinical trial (NCT01330966), pazopanib was evaluated in patients with unresectable and metastatic conventional CS, achieving a disease control rate of 43%. A median progression-free survival (PFS) of 7 months, and a median overall survival of 17 months was also achieved [141]. Similarly, regorafenib, another VEGFR-targeting agent, demonstrated the ability to prolong PFS in patients with advanced CS (Table 2) [142].

These findings highlight the relevance of VEGFRs and other angiogenic pathways in CS pathophysiology and support further exploration of anti-angiogenic therapies. Additionally, combining anti-angiogenic agents with immune checkpoint inhibitors and chemotherapy has been proposed as a promising strategy to enhance therapeutic outcomes in CS [143].

## 10. Immune-Based Therapies for Chondrosarcoma

Emerging immunotherapies have shown considerable success in various cancers, and recent studies are exploring their potential in CS. Among immunogenic targets, cancer testis antigens (CTAs) such as MAGE, NY-ESO-1, TRAG-3/CSAGE, and PRAME have demonstrated promise in CS [144]. In some studies, CD8^+^ T cells were able to lyse CS cells expressing these CTAs, indicating the potential for antigen-specific T cell-based therapies [144,145,146].

Immune checkpoint proteins have also been investigated in CS, particularly programmed cell death protein 1 (PD-1) and its ligand PD-L1. PD-1 expression is increased in CS tissues compared to normal bone, and PD-L1 expression varies by CS subtype—one study reported PD-L1 positivity in 41% of dedifferentiated CS samples [147,148]. In a cohort of 59 conventional CS samples, PD-L1 and PD-L2 expression rates were 67% and 42%, respectively, with PD-L1 correlating with higher tumor grade and recurrence risk [44]. Despite these findings, clinical investigation of PD-1/PD-L1 blockade in CS remains limited. In the SARC028 clinical trial (NCT02301039), one of five CS patients exhibited an objective response to the PD-1 inhibitor pembrolizumab (Table 2) [45]. A separate case study described a 74-year-old patient with dedifferentiated CS who achieved a partial response following six cycles of nivolumab (anti-PD-1) [149]. More recently, CAR-T cell therapies, successful in hematologic malignancies, have shown preclinical potential in CS and are being explored clinically (Table 2) [150].

**Figure 2:**
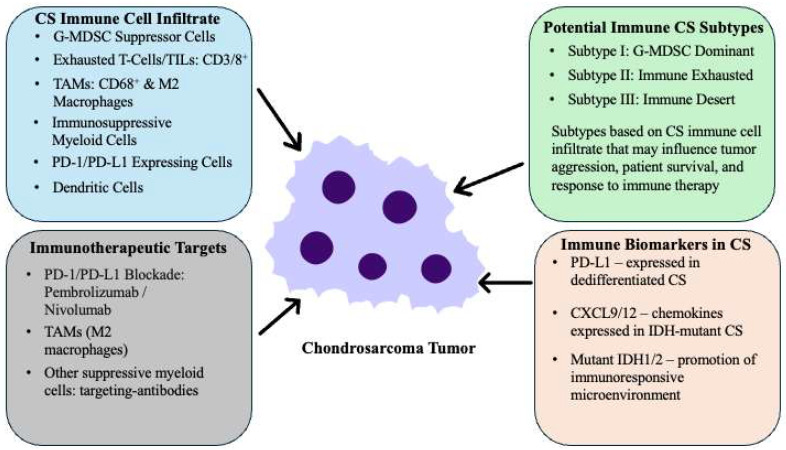
Tumor Immune Microenvironment and Immunotherapeutic Strategies in Chondrosarcoma. This schematic illustrates the immune microenvironment of CS, depicting key immune cell populations including granulocytic myeloid-derived suppressor cells (G-MDSCs), tumor-associated macrophages (TAMs), dendritic cells, and exhausted T cells. CS tumors can be stratified into three potential immune subtypes based on infiltrate composition: Subtype I (G-MDSC dominant), Subtype II (immune exhausted), and Subtype III (immune desert), each associated with varying degrees of immune suppression and therapy response. Biomarkers such as PD-L1, CXCL9/12, and mutant IDH1/2 are highlighted for their roles in shaping the immune landscape. Notably, PD-L1 is expressed predominantly in dedifferentiated CS, while IDH1/2 mutations are linked to increased expression of CXCL9/12 and a more immunoresponsive microenvironment. Therapeutic strategies shown include PD-1/PD-L1 immune checkpoint inhibitors (e.g., pembrolizumab, nivolumab), M2-polarized TAMs, and other immunosuppressive myeloid cells. This figure emphasizes the potential of biomarker-guided immunotherapies in overcoming the immune evasion characteristic of CS tumors.

Recent studies suggest that the immune cell composition of the CS tumor microenvironment may influence aggressiveness, survival, and immunotherapy response (Figure 2). A multi-omic profiling study of 20 conventional CS cases proposed an immune-based classification comprising three subtypes: (I) “G-MDSC dominant,” characterized by granulocytic myeloid-derived suppressor cells; (II) “immune exhausted,” featuring exhausted T cells and dendritic cells; and (III) “immune desert,” with minimal immune infiltration [151]. Notably, 60% of cases fell into the immune desert category, with sparse macrophages, monocytes, NK cells, and B cells. Although previous studies suggested no link between IDH mutation status and immune infiltration, this analysis found that IDH1/2-mutant CS tumors were more commonly associated with immune-enriched (types I and II) profiles, characterized by elevated chemokines such as CXCL9 and CXCL12. In a subset of 12 CS patients treated with PD-1 inhibitors (pembrolizumab or sintilimab), clinical benefit—partial response or stable disease—was observed only in those classified within the immune exhausted subtype [151].

Other studies have highlighted macrophages as the predominant immune cell type in the CS microenvironment, comprising up to 75% of infiltrating leukocytes [97,152]. In dedifferentiated CS, tumor-infiltrating lymphocytes (TILs) and tumor-associated macrophages (TAMs) exhibit divergent prognostic associations: higher CD3^+^ and CD8^+^ T cell densities correlate with improved overall survival, whereas a higher CD68^+^ macrophage to CD8^+^ T cell ratio and elevated macrophage-attracting chemokines indicate poorer prognosis. TAMs are typically peritumoral in conventional CS but are more abundant within dedifferentiated regions of dedifferentiated CS [152]. Patients with metastatic disease at diagnosis also exhibit greater infiltration of immunosuppressive, M2-polarized TAMs, marked by CD68^+^ and CD163^+^ expression [152]. These observations reinforce the complex immunosuppressive architecture of the CS microenvironment and its potential implications for patient outcomes.

Collectively, these findings underscore the prognostic and therapeutic relevance of immune profiling in CS. Detailed characterization of the immune landscape may guide the application of immunotherapeutic strategies, particularly immune checkpoint blockade, in this otherwise treatment-resistant malignancy.

## 11. Epigenetic-Based Therapies for Chondrosarcoma

Mutations in IDH1/2 contribute to increased DNA and histone methylation in chondrosarcoma (CS), although the resulting epigenetic landscape varies by subtype. Specifically, IDH-mutant dedifferentiated CS exhibits lower levels of hypermethylation and distinct methylated loci compared to IDH-mutant conventional CS [130]. These insights have spurred interest in epigenetic therapies as a potential treatment strategy for CS.

Two primary classes of epigenetic drugs are under investigation: DNA methyltransferase (DNMT) inhibitors, such as HDAC inhibitors, including vorinostat, romidepsin, belinostat, and panobinostat (Table 2) [11]. In preclinical models, combining 5-aza with vorinostat demonstrated potent antitumor effects both in vitro and in vivo using the JJ012 cell line-derived xenograft model, outperforming either agent alone. This combination enhanced DNA damage, induced interferon-stimulated genes, increased PD-L1 expression, and stimulated the innate immune response [153].

Building on these findings, a phase II clinical trial (NCT04340843) evaluated the DNMT inhibitor guadecitabine (SGI-110) in combination with the HDAC inhibitor belinostat. This combination was observed in patients with unresectable or metastatic conventional CS. However, the trial did not meet its primary endpoint of overall response rate (ORR) [154]. Current research is now focused on combinatorial approaches that integrate epigenetic therapy, immunotherapy, and chemotherapy to improve therapeutic outcomes [153,155].

### Metabolic Alterations and Potential Targets in Chondrosarcoma

Malignant tumor cells often exhibit metabolic adaptations that enable survival and proliferation in nutrient-poor and hypoxic tumor microenvironments. One prominent example is the Warburg effect, or aerobic glycolysis, wherein tumor cells preferentially utilize glycolysis even in the presence of oxygen. An elevated rate of aerobic glycolysis is a common metabolic feature across many sarcomas. In CS, metabolic alterations are linked to both the epigenetic landscape and the tumor microenvironment, with progression to higher tumor grades being accompanied by upregulation of glycolytic genes [156,157]. Notably, inhibition of glycolysis has been shown to help overcome drug resistance, positioning glucose metabolism pathways as potential therapeutic targets in CS.

Moreover, mutant and non-mutant IDH CSs exhibit distinct metabolic profiles. Mutant IDH CSs display elevated lactate levels and increased late tricarboxylic acid (TCA) cycle intermediates, indicating enhanced glycolytic metabolism and possible anaplerotic influx into the TCA cycle. Prior gene expression profiling corroborates this metabolic shift, demonstrating increased expression of several metabolic genes in mutant IDH CSs [120].

Additionally, CS cells can sustain growth under glucose starvation by accumulating NAD^+^ and activating the SIRT1–HIF-2α axis. Inhibition of SIRT1 diminishes HIF-2α transcriptional activity and re-sensitizes CS cells to doxorubicin-induced cytotoxicity, revealing another exploitable metabolic vulnerability in CS [31].

## 12. Conclusions

CS remains one of the most challenging bone malignancies due to its inherent resistance to chemotherapy and radiotherapy. This resistance stems in part from the tumor’s complex molecular heterogeneity, its uniquely ECM-rich TME, and the lack of validated biomarkers and actionable targets. However, recent advances in genomic and epigenomic profiling have uncovered recurrent genetic alterations in CS, including copy number variations (CNVs) involving MYC, CDK4, and CDKN2A; mutations in IDH1/2, TP53, and COL2A1; and fusion genes such as HEY1-NCOA2 and IRF2BP2-CDX1, alongside key epigenetic and metabolic changes. These discoveries have expanded the repertoire of potential diagnostic markers and therapeutic targets in CS.

Integrated multi-omics approaches, coupled with improved preclinical models—including 3D cultures, organoids, and xenograft systems—are deepening our understanding of CS tumor biology and its interactions with the microenvironment. Promising therapeutic strategies now include IDH inhibitors, HDAC inhibitors, CDK4 inhibitors, HIF-2α antagonists, DR5 agonists, and emerging immunotherapies, particularly for biomarker-defined patient subgroups. Although clinical translation has been slow, especially in conventional CS, recent trials indicate that select CS subtypes may benefit from personalized, biomarker-driven treatments.

Overall, the molecular landscape of CS is becoming increasingly well-defined, paving the way for the development of more effective, targeted, and durable therapies. Continued research into the interplay between genomic alterations, epigenetic regulation, and the immune microenvironment will be critical for overcoming therapeutic resistance and improving clinical outcomes for patients afflicted by this devastating disease.

## Figures and Tables

**Table 1:** Genetic Alterations Observed in Chondrosarcoma.

Genetic Alteration	Frequency	CS Subtype(s)	Functional Impact	Therapeutic Implications
IDH1/2 mutations	~80%	Conventional, Dedifferentiated	Epigenetic reprogramming via D-2HG accumulation	Targeted by IDH inhibitors
TP53 mutations	~25–30% (high-grade)	Dedifferentiated	Loss of tumor suppressor function, genomic instability	Potential MDM2 inhibitors
PTEN Deletion	Associated with a TP53 mutation	Recurrent dedifferentiated	Increased proliferative index	Research is ongoing
COL2A1 mutations	Frequent in conventional CS	Conventional	ECM dysregulation and excess type II collagen	Research is ongoing
HEY1–NCOA2 fusion	~85% in mesenchymal CS	Mesenchymal	Oncogenic transcriptional program activation	Targeting fusion-specific pathways (research ongoing)
IRF2BP2-CDX1 fusion	Rare	Mesenchymal	Tumorigenesis likely via TP53 and IRF2	Research is ongoing
CDK4 amplification	Common in high-grade and dedifferentiated CS	High-grade and dedifferentiated	Cell cycle dysregulation via inhibition of the Rb pathway	CDK4 inhibitors (e.g., Palbociclib)
RB1 deletion	Common in high-grade and dedifferentiated CS	High-grade and dedifferentiated	Loss of cell cycle progression regulation	CDK4 inhibitors (e.g., Palbociclib); Research is ongoing
MYC amplification	Detected in high-grade CS	High-grade	Promotes proliferation and survival	MYC-targeted strategies (experimental)
CDKN2A deletion	Common in dedifferentiated CS	Dedifferentiated	Loss of cell cycle checkpoint control	Potential for CDK4/6 inhibitors
SAS amplification	Rare	High-grade and dedifferentiated	Tumorigenesis via an unknown mechanism	Research is ongoing
GLI amplification	Rare	Conventional and dedifferentiated	Enhances survival and differentiation via the HH pathway activation	HH pathways inhibitors (e.g., Vismodegib)
PTHLH amplification	Observed in high-grade CS	Dedifferentiated	Promotes chondrocyte growth, differentiation, and possibly tumor progression	Research is ongoing
PPFIBP1 amplification	Observed in high-grade CS	Dedifferentiated	Invasiveness and metastasis via S100A4 upregulation	Research is ongoing
INK4A/INK4A-ARF deletion	Common in dedifferentiated CS	Dedifferentiated	Loss of tumor suppressors CDKN2A/CDKN2C and p16, results in unchecked cell cycle progression	Potential for CDK4/6 inhibitors
RPS6 deletion	Rare	Dedifferentiated	Disrupts cell growth regulation through the ribosomal protein pathway	Research is ongoing
EXTL2 deletion	Observed in secondary peripheral CS	Secondary peripheral	Contributes to osteochondroma transformation into secondary peripheral CS	Research is ongoing
EXT1/2 mutations	Observed in secondary peripheral CS	Secondary peripheral	Impaired heparan sulfate biosynthesis; Facilitates osteochondroma transformation into secondary peripheral CS	Research is ongoing
EPAS1 amplification	Reported in high-grade CS	High-grade	Promotes drug resistance via HIF-2α	HIF-2α inhibitors (e.g., PT2385, PT2977)
SIRT1	Highly expressed in high-grade and dedifferentiated CS	High-grade and dedifferentiated	HIF-2α activation, metastasis	Research is ongoing
Hedgehog mutations	Rare	Conventional and dedifferentiated	Activates GLI TFs, enhancing survival and differentiation	HH pathway inhibitors (e.g., Vismodegib)

**Table 2:** Therapeutic Agents Under Investigation in Chondrosarcoma.

Therapeutic Class	Agent(s)	Target	Preclinical/Clinical Status	CS Subtype/Context
IDH Inhibitors	Ivosidenib, Enasidenib	IDH1/2 mutations	Phase I/II/III clinical trials in IDH-mutant tumors (NCT04278781; NCT04278781; NCT06127407)	IDH1/2-mutant conventional and dedifferentiated CS
HDAC Inhibitors	Panobinostat, Belinostat, Vorinostat, Romidepsin	Epigenetic dysregulation (histone deacetylation)	Preclinical; Phase II clinical trial (NCT04340843)	Conventional and dedifferentiated CS; unresectable or metastatic conventional CS
DMNT Inhibitors	5-aza-2′-deoxycytidine, Decitabine, Guadecitabine	Epigenetic dysregulation (DNA methyltransferase)	Phase II clinical trial (NCT04340843)	Unresectable or metastatic conventional CS
CDK4/6 Inhibitors	Palbociclib	CDK4 amplification, pRB pathway	Preclinical evidence in CS	Dedifferentiated CS
HIF-2α Inhibitors	TC-S7009	HIF-2α	Preclinical	High-grade CS expressing HIF-2α
SIRT1 Inhibitors	EX527	SIRT1	Preclinical	Conventional CS
Immune Checkpoint Inhibitors	Pembrolizumab, Nivolumab, Sintilimab	PD-1/PD-L1 axis	Clinical trial (NCT02301039); in vivo study	Dedifferentiated and immune-enriched CS subtypes
CAR-T Cells	CSPG4-directed CAR-T cells	CSPG4 antigen	Preclinical efficacy demonstrated in CS models	CSPG4-expressing CS
TRAIL Receptor Agonists	TRAIL/DR5 agonists (e.g., Dulanermin)	TRAIL receptors, e.g., DR5	Preclinical studies	Metastasis CS patient
DR5	DR5 Ab agonists, e.g., anti-DR5 IgM, INBRX-109	DR5	Phase I/II clinical trials (NCT04553692; NCT03715933; NCT03277924; NCT04950075)	Conventional CS; unresectable or metastatic conventional CS
Hedgehog Pathway Inhibitors	HPI-4, IPI-929	SMO; Hedgehog-GLI signaling	Preclinical	Conventional CS
VEGFR Inhibitors	Pazopanib, Regorafenib	VEGFRs	Phase II clinical trial: NCT01330966	Unresectable and metastatic conventional CS

## Abbreviations

aKG, Alpha-Ketoglutarate; CAR-T, Chimeric Antigen Receptor T cell; CDX, Cell Line-Derived Xenograft; CGH, Comparative Genomic Hybridization; CNV, Copy Number Variation; COL2A1, Collagen Type II Alpha 1 Chain; CS, Chondrosarcoma; CTA, Cancer Testis Antigen; D-2HG, D-2-Hydroxyglutarate; DNMT, DNA Methyltransferase; DR5, Death Receptor 5; ECM, Extracellular Matrix; FOXO, Forkhead Box O; FDA, Food and Drug Administration; G-MDSC, Granulocytic Myeloid-Derived Suppressor Cell; GSEA, Gene Set Enrichment Analysis; HDAC, Histone Deacetylase; HEY1, Hairy/Enhancer-of-Split Related with YRPW Motif Protein 1; HIF-1α, Hypoxia-Inducible Factor 1 Alpha; HIF-2α, Hypoxia-Inducible Factor 2 Alpha; IDH, Isocitrate Dehydrogenase; lncRNA, Long Non-Coding RNA; LOH, Loss of Heterozygosity; MDSC, Myeloid-Derived Suppressor Cell; miRNA, MicroRNA; MDM2, Mouse Double Minute 2 Homolog; MSC, Mesenchymal Chondrosarcoma; NGS, Next-Generation Sequencing; ORR, Overall Response Rate; PDX, Patient-Derived Xenograft; PD-1, Programmed Cell Death Protein 1; PD-L1, Programmed Death-Ligand 1; PDGFR, Platelet-Derived Growth Factor Receptor; PFS, Progression-Free Survival; PTCH1, Patched 1; RT-qPCR, Real-Time Quantitative Polymerase Chain Reaction; SIRT1, Sirtuin 1; SMO, Smoothened; SUFU, Suppressor of Fused; TAM, Tumor-Associated Macrophage; TCA, Tricarboxylic Acid Cycle; TIL, Tumor-Infiltrating Lymphocyte; TME, Tumor Microenvironment; TRAIL, Tumor Necrosis Factor-Related Apoptosis-Inducing Ligand; VEGF-A, Vascular Endothelial Growth Factor A; VEGFR, Vascular Endothelial Growth Factor Receptor; WES, Whole-Exome Sequencing.
